# Nanostructured carbon materials decorated with organophosphorus moieties: synthesis and application

**DOI:** 10.3762/bjnano.8.52

**Published:** 2017-02-22

**Authors:** Giacomo Biagiotti, Vittoria Langè, Cristina Ligi, Stefano Caporali, Maurizio Muniz-Miranda, Anna Flis, K Michał Pietrusiewicz, Giacomo Ghini, Alberto Brandi, Stefano Cicchi

**Affiliations:** 1 Dipartimento di Chimica Ugo Schiff Università di Firenze, Via della Lastruccia 3–13, 50019 Sesto Fiorentino, Italy; 2 Consorzio Interuniversitario Nazionale per la Scienza e Tecnologia di Materiali INSTM, 50123 Firenze, Italy; 3 Istituto dei Sistemi Complessi Consiglio Nazionale delle Ricerche, 50019 Sesto Fiorentino, Italy; 4 Department of Organic Chemistry Maria Curie-Skłodowska University, ul. Gliniana 33, 20-614 Lublin, Poland; 5 Nanesa S.r.l. Via Setteponti 143 - 1, 52100 Arezzo, Italy

**Keywords:** azides, click chemistry, heterogeneous catalysis, organocatalysis, phosphorus

## Abstract

A new synthetic approach for the production of carbon nanomaterials (CNM) decorated with organophosphorus moieties is presented. Three different triphenylphosphine oxide (TPPO) derivatives were used to decorate oxidized multiwalled carbon nanotubes (ox-MWCNTs) and graphene platelets (GPs). The TPPOs chosen bear functional groups able to react with the CNMs by Tour reaction (an amino group), nitrene cycloaddition (an azido group) or CuAAC reaction (one terminal C–C triple bond). All the adducts were characterized by FTIR, Raman spectroscopy, TEM, XPS, elemental analysis and ICP-AES. The cycloaddition of nitrene provided the higher loading on ox-MWCNTs and GPs as well, while the Tour approach gave best results with nanotubes (CNTs). Finally, we investigated the possibility to reduce the TPPO functionalized CNMs to the corresponding phosphine derivatives and applied one of the materials produced as heterogeneous organocatalyst in a Staudinger ligation reaction.

## Introduction

**Figure 1:**
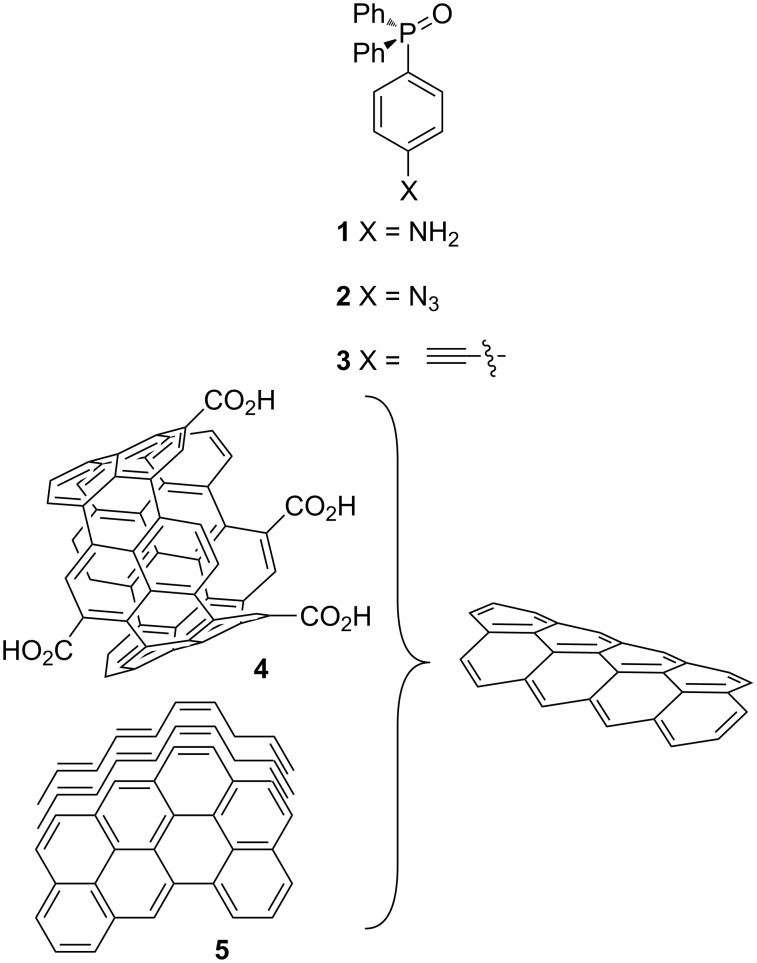
Structure of phosphine oxide derivatives **1**–**3**, of ox-MWCNTs **4** and of GPs **5**.

The term of carbon nanomaterial (CNMs) comprises many different allotropic species of carbon, fullerene, carbon nanotubes and graphene being the most studied and used. Since their discovery [1–3], they have become very attractive for researchers, due to their peculiar physical and chemical proprieties such as chemical and thermal stability, electronic conductivity, and their nanometric dimensions that prompted their application in chemistry of materials [4]. Furthermore, their functionalization with an increasing number of molecular moieties [5–6] has extended their use in new fields ranging from biology [7] to catalysis. Despite such variability, the classes of reactions most used for their functionalization are the same and limited in number. The Tour reaction [8] is one of the most used synthetic approach for the functionalization of CNTs and GPs. In this reaction, an aniline derivative is transformed into a diazonium ion that, upon decomposition and reduction, affords a radical species, responsible for the functionalization of the graphitic surface [9]. A useful alternative to this approach is the reaction of azido derivative with CNMs: the high temperature required for the process decomposes the azido group to a reactive nitrene species that react with the graphitic surface to form an aziridine ring [10]. Finally, the use of the CuAAC reaction [11], between an azide group and a terminal alkyne, has revealed a practical synthetic approach for the decoration of CNMs with a variety of molecular moieties [12–13]. In this work, we present our results in the functionalization of oxidized MWCNTs **4** and multilayer graphene platelets (GPs) **5** [14] using amino- or azido-functionalized triphenylphosphine oxides **1** and **2** and the terminal alkyne **3** (see Figure 1).

Such work led to the production of phosphine oxide substituted CNMs. A limited number of works describe the introduction of phosphorous moieties onto CNMs. Muleja et al. synthesized a MWCNTs-TPP system modifying the nanotube with the introduction of a phenyl bromide group via diazonium coupling and then of the phosphinating reagent [15]. Hamilton et al. described the functionalization of carbon nanotube with triphenylphosphine oxide (TPPO) using the carboxylic group, introduced with the oxidation, to covalently link the TPPO [16]. Only one example describes the functionalization of a graphene based material with a phosphine, the authors report the synthesis, characterization and test of palladium nanoparticles supported on phosphine decorated graphene oxide [17]. The interest in the introduction of a phosphine oxide group in CNMs is due to its ability to promote a wide varieties of chemical transformation [18]. Phosphines have found large application in organocatalytic processes [19–20] and, recently, also triphenylphosphine oxide (TPPO) have found similar application [21–24]. Despite their utility, very few examples of heterogeneous catalysts are described. One example was reported by Tang, who developed a phosphine oxide derivative linked to a polystyrene resin [25]. For this reason, a new class of phosphine oxide functionalized CNMs can be of interest. Not to say of the wide possibilities offered by the production of metal nanohybrid upon complexation of metal nanoparticles or metal ions by the phosphine functionalities [26].

## Results and Discussion

**Scheme 1:**
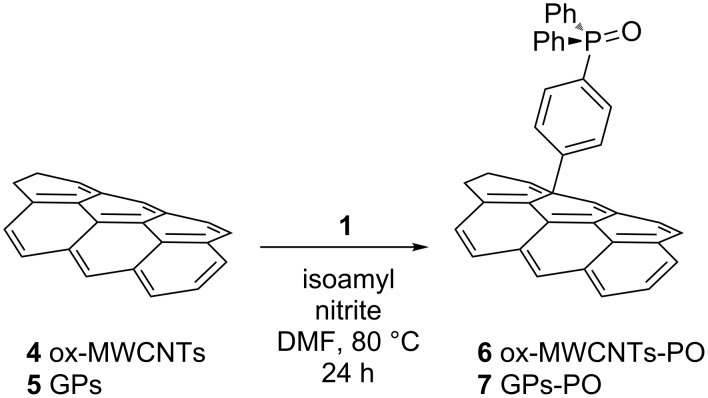
The Tour reaction applied on CNMs **4** and **5**.

The CNMs substrates used for this study are oxidized MWCNTs **4** and GPs **5**. The oxidation of pristine MWCNTs [27–28] afforded an easily dispersible material and removed any possible metal impurities present in the starting substrate. GPs were used as example of easily accessible and low cost graphitic material (see Experimental section). The first functionalization studied was the reaction of (4-aminophenyl)diphenylphosphine oxide (**1**) with the two substrates **4** and **5** (Scheme 1).

This reaction was carried out using the well described Tour protocol [29–30]: the nanomaterials and **1** were dispersed in DMF, then isopentyl nitrite was added and the mixture kept at 80 °C for 24 h. The ox-MWCNTs derivative **6** was isolated through filtration over a 0.2 µm PTFE membranes followed by repeated washings with different solvents to remove excess reagents, while the GPs **7** was recovered after several cycles of centrifugation and dispersion.

**Scheme 2:**
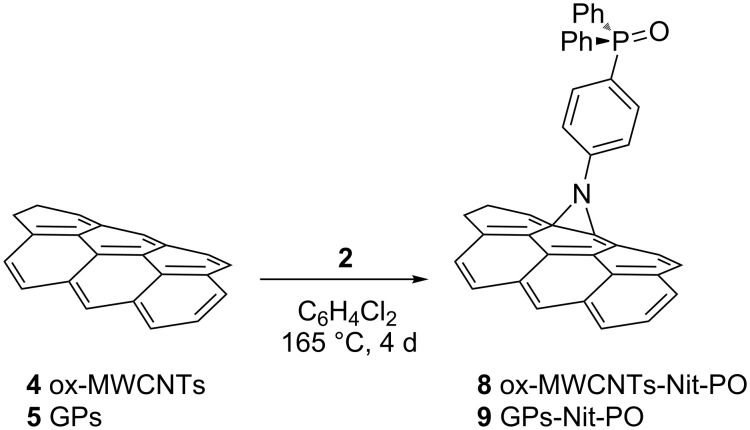
The [1 + 2] nitrene cycloaddition on CNMs **4** and **5**.

Subsequently, substrates **4** and **5** were reacted with the azido derivative **2** (Scheme 2).

The mixture of the reagents in dichlorobenzene was kept in the ultrasound bath for 25 min, to obtain a homogeneous dispersion, and subsequently stirred at 165 °C for four days [10,31–32]. Again CNTs derivative **8** was more easily isolated by filtration and repeated washing for complete removal of the excess reagents while GPs-Nit-PO derivative **9** was recovered using cycles of centrifugation and re-dispersion of the carbon material in a 1:1 isopropyl ether-isopropanol solution.

**Scheme 3:**
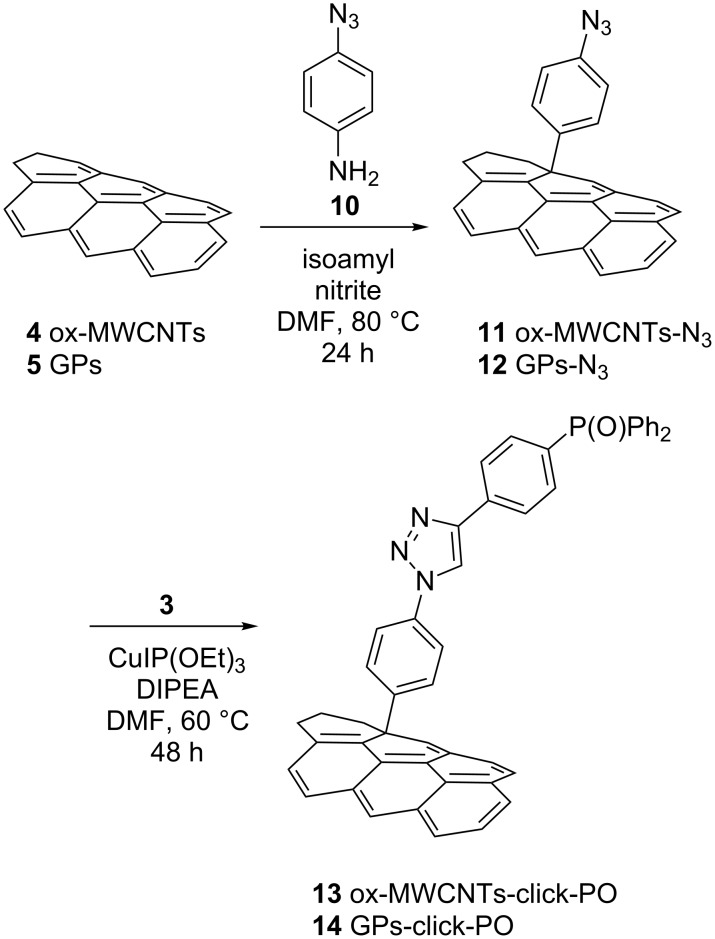
The Tour reaction with 4-azido aniline (**10**) on CNMs **4** and **5** and the subsequent CuAAC reaction.

The decoration of the carbonaceous substrates, **4** and **5**, with (4-ethynylphenyl)diphenylphosphine oxide (**3**), via the CuAAC reaction, required their previous modification with the introduction of azido groups. For this purpose, CNMs **4** and **5** were reacted with 4-azidoaniline (**10**) following, again, the Tour protocol affording compounds **11** and **12** (Scheme 3) [33].

The successful decoration of NMs **11** and **12** was confirmed by elemental analysis and IR spectroscopy (see Supporting Information File 1, Figures S9 and S10) and, finally, they were reacted with compound **3** to afford the functionalized compounds **13** and **14** (Scheme 3).

The characterization of each material produced was performed via elemental analysis, inductively coupled ion plasma atomic emission spectroscopy (ICP-AES) analysis, IR and Raman spectroscopy, XPS analysis. Elemental and ICP analyses were useful for the determination of the loading after each chemical transformation. For example, the % content of nitrogen in compounds **8** (1.32%) and **9** (0.42%) suggested a loading degree for **8** (0.58 mmol/g) and **9** (0.3 mmol/g). The values obtained for **11** and **12** indicated a functionalization degree of 1.63 mmol/g and 0.075 mmol/g, respectively. The presence of the azido group in **11** and **12** was confirmed by the signal at 2118 cm^−1^ in the FTIR spectra (see Supporting Information File 1, Figures S9 and S10). The ICP-AES was used to determine the amount of phosphorus in the complex matrix. The samples were previously mineralized by treatment with nitric acid and a hydrogen peroxide solution at high temperature in microwave. The data obtained are reported in Table 1.

**Table 1:** ICP-Analysis results and P loading.

Entry	Compound	ICP-AES P %	mmol/g P

1	**4**	–	–
2	**5**	–	–
3	**6**	1.26	0.40
4	**7**	0.27	0.09
5	**8**	1.81	0.58
6	**9**	0.61	0.20
7	**13**	0.85	0.27
8	**14**	0.06	0.02

From the data reported in Table 1 it is evident the higher reactivity of ox-MWCNTs **4** (entries 3, 5 and 7) respect to GPs **5** (entries 4, 6 and 8) as it was expected considering the different nature of the two substrates [34]. For both series of reactions the higher efficiency was found for the nitrene cycloaddition (Table 1, entries 5 and 6) followed by the Tour reaction (Table 1, entries 3 and 4). The decoration using the CuAAC reaction (Table 1, entries 7 and 8) revealed the less efficient. To be noted that this is not due to a poor content in the azido component (see data for compounds **11** and **12**) but to the low reactivity found in the CuAAC step. To be noted is the good agreement for the loading values obtained with the elemental analysis (see earlier) and with the ICP AES analysis for compound **8** and **9** (Table 1, entries 5 and 6).

**Figure 2:**
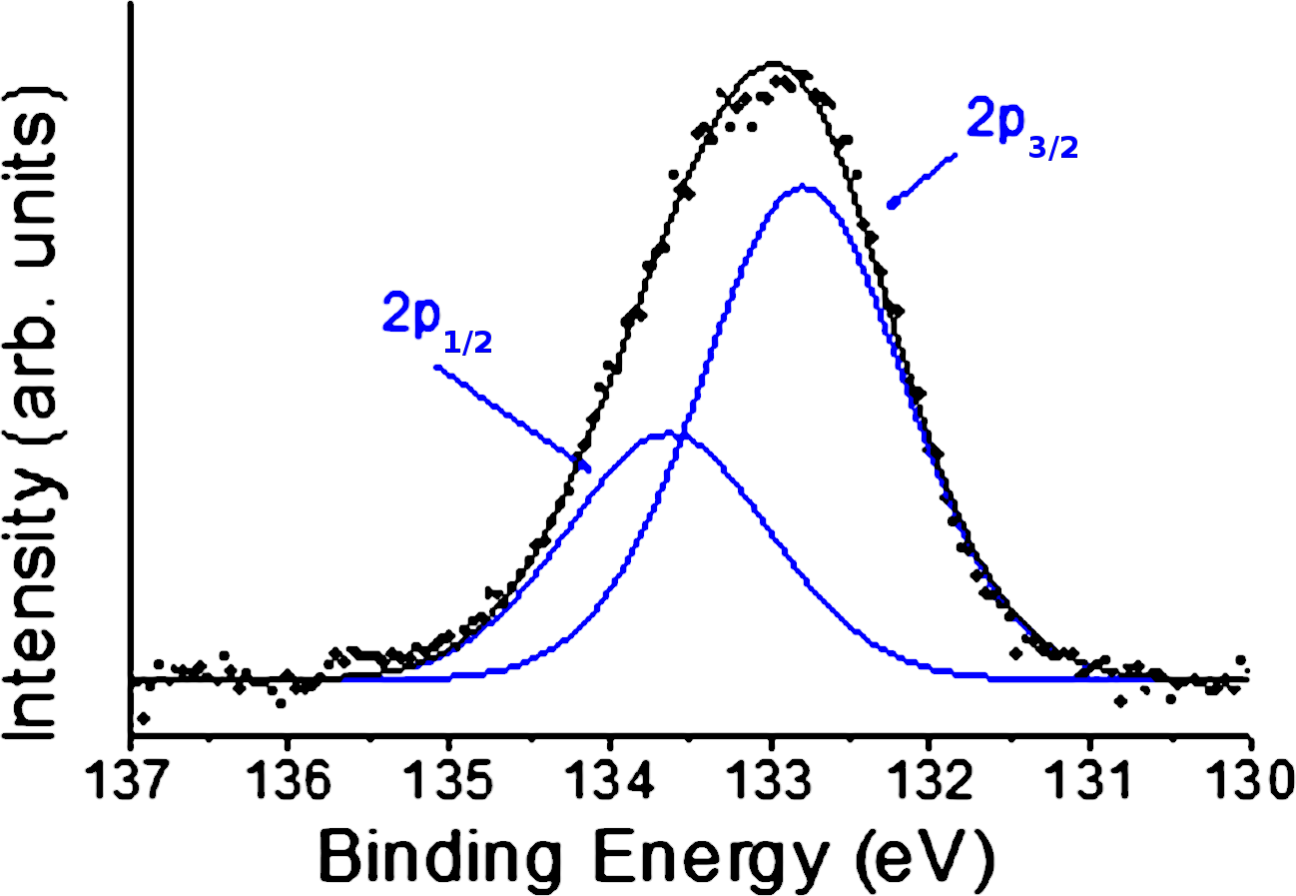
Fitting of the XPS spectrum characteristic of P collected on GPs-Nit-PO **9** showing the two components (2p_1/2_ and 2p_3/2_) relative to the phosphine oxide group (for XPS spectra of the other compounds see Supporting Information File 1).

X-ray photoelectron spectroscopy analysis showed the presence of the P(V) atoms in all the samples considered. The samples for the analysis where prepared by dispersion of 1 mg of substance in 1 mL of isopropanol and the dispersion was drop casted on a cleaned glass support. The spectra of all TPPO decorated materials were recorded and all showed a signal at a binding energy of 132.8 eV, where the two component 2p^3/2^ and 2p^1/2^, compatible with a phosphine oxide species, can be observed (see Figure 2).

**Figure 3:**
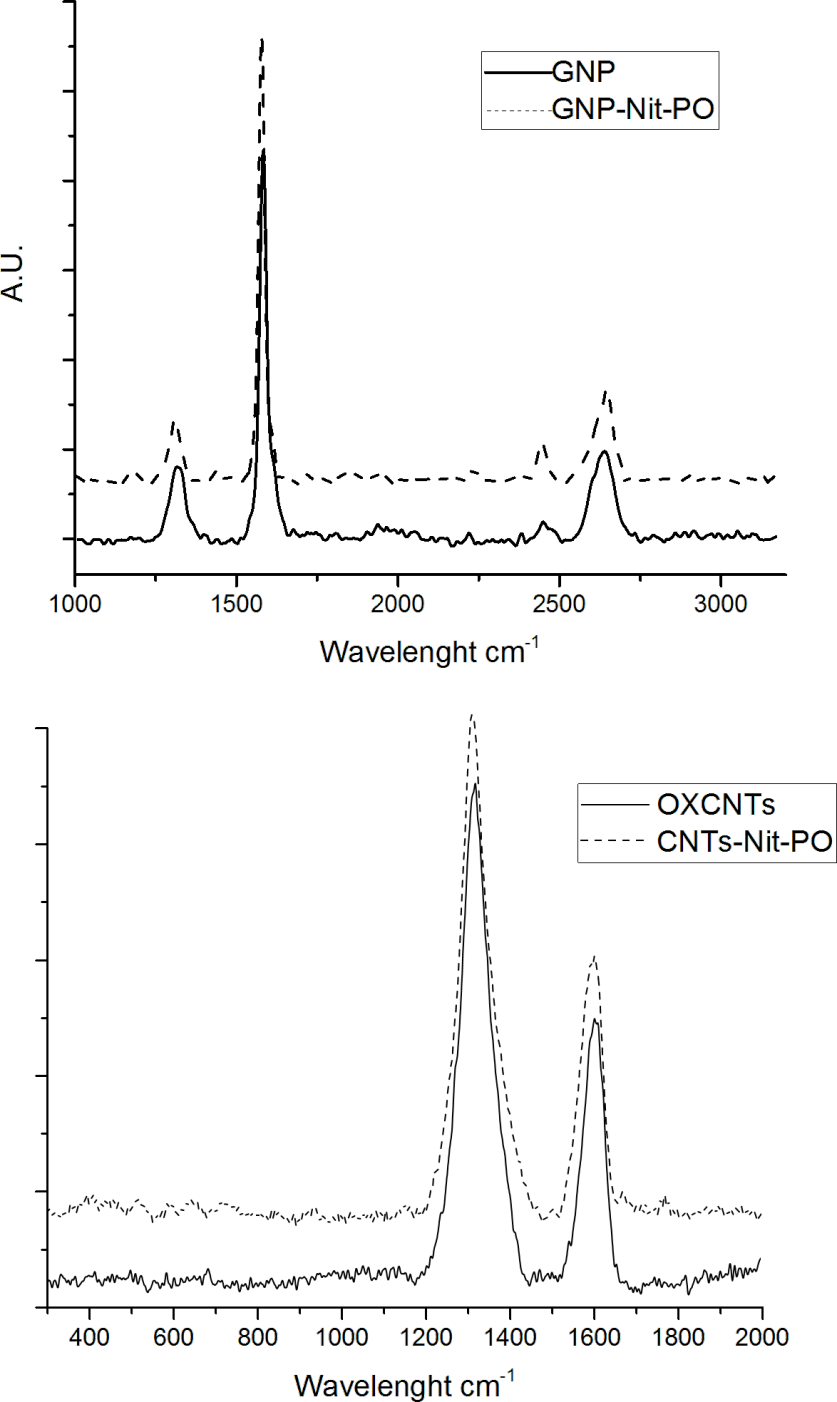
Raman spectra: GPs **5** vs GPs-Nit-PO **9** (top), ox-MWCNTs **4** vs ox-MWCNTs-Nit-PO **8** (bottom).

Raman spectroscopy analyses were performed on the most functionalized samples, compound **8** and **9** (see Figure 3). Generally, CNMs show two main bands in their Raman spectra: one at ≈1580 cm^−1^ (G band) related to sp^2^ graphitic domain and the second at ≈1360 cm^−1^ (D band) attributed to the amorphous carbon or deformation vibrations of a hexagonal ring [15]. Raman spectra of ox-MWCNTs **4** (Figure 3, bottom) showed the D and G bands centered at 1320 and 1607 cm^−1^, respectively [35], while for compound **8** the band were centered at 1312 and 1590 cm^−1^. Despite the ox-MWCNTs **4** already showed an intense D band (*I*_D_/*I*_G_ = 2.57), the functionalization further increased the D band intensity, so that the *I*_D_/*I*_G_ for compound **8** raised to 3.58. The Raman characterization of the GPs **5** (Figure 2, top) showed the D and G bands at 1320 and 1580 cm^−1^ with a visible shoulder at 1610 cm^−1^, while at 2640 cm^−1^ is visible the overtone band 2D typical of graphene. This latter band, sharp and intense in monolayer graphene, is broadened confirming the high number of layers of the GPs. Upon functionalization The GPs-Nit-PO **9** spectrum showed the same bands with no significant differences respect to **5**.

**Figure 4:**
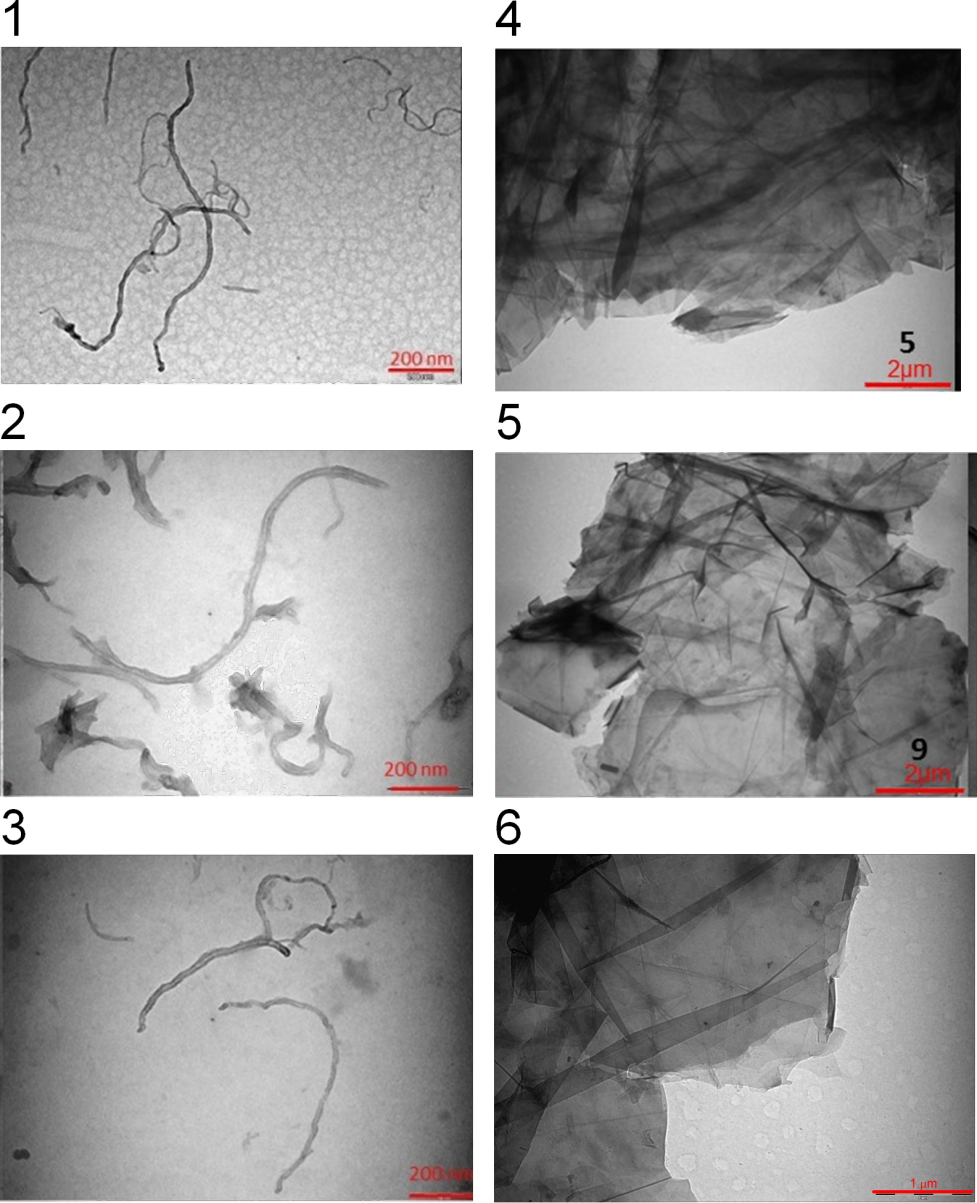
TEM images of ox-MWCNTs **4** (1), ox-MWCNTs-PO **6** (2), ox-MWCNTs-Nit-PO **8** (3), GPs **5** (4) GPs-PO **7** (5), GPs-Nit-PO **9** (6).

TEM images of functionalized CNM are shown in Figure 4. No significant difference can be found in the morphology of the materials. In particular, as confirmed by the Raman analysis, the GPs present a multilayer structure and no further exfoliation of the multi-layer GPs was observed.

**Scheme 4:**
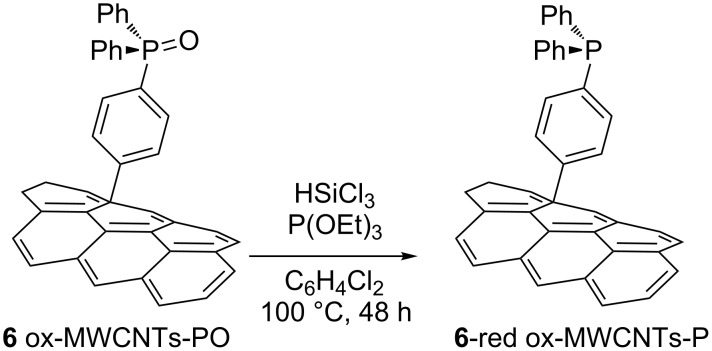
Reduction of phosphine oxide **6** to the corresponding phosphine **6**-red.

A useful extension of these synthetic approaches is the chance to reduce the P=O moiety to the corresponding phosphine. The phosphorus-phosphorus, trichlorosilane mediated oxygen transfer protocol, developed by Hamilton [16] and Wu [36], was used with compound **6** (Scheme 4). The reaction was carried out in a Pyrex tube, heating for 48 h, a degassed solvent solution of compound **6**, trichlorosilane and triethyl phosphite as final oxygen acceptor. The reduction of the phosphine oxide moiety was followed by XPS analysis and confirmed by FTIR spectroscopy.

**Figure 5:**
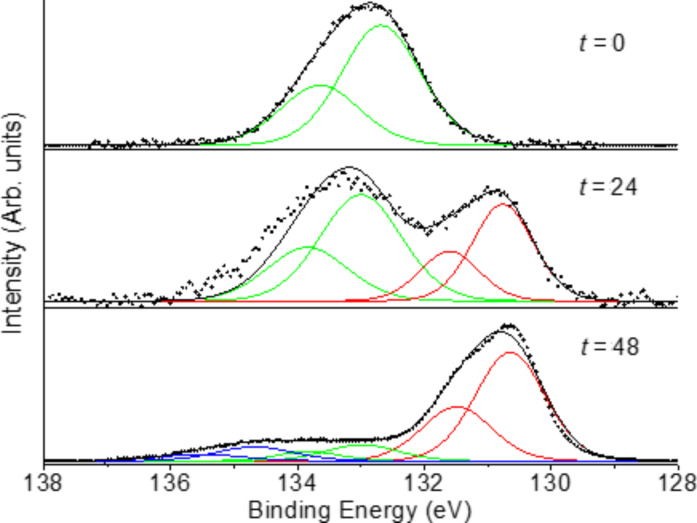
XPS analysis of samples form the reduction reaction of compound **6**: starting material (top), after 24 h (middle), after 48 h (bottom).

Figure 5 shows the XPS spectra registered on starting material **6** (*t* = 0), and of the reaction product after 24 h and after 48 h. The XPS analysis of the starting material showed only the peak at binding energy 132.8 eV (related to presence the phosphine oxide group), after 24 h a new peak, related to the reduced phosphorus atoms, appeared at 130.8 eV, accordingly with value reported by Swartz et al. [37]. After 48 h the peak at 130.8 eV is the main one showing that the reaction is almost complete.

The FTIR spectroscopy confirmed the reduction of the phosphine oxide group with the disappearance of the band at 1114 cm^−1^ related to the P=O stretching vibration (Supporting Information File 1, Figures S2–S4).

**Scheme 5:**
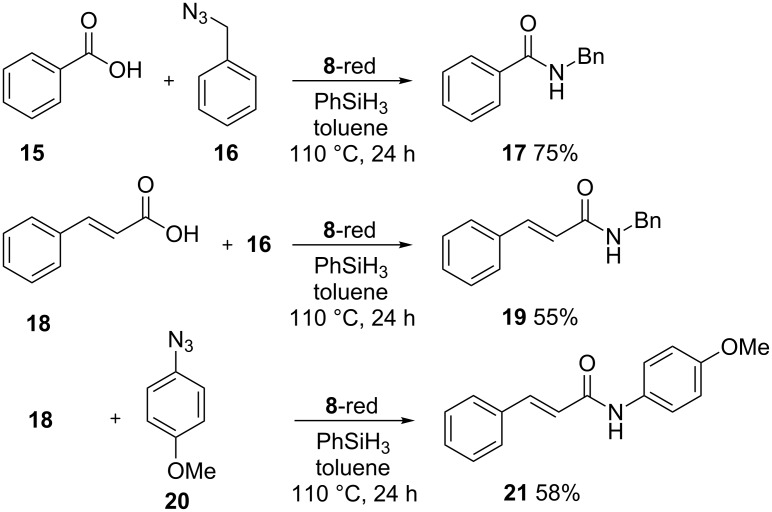
The Staudinger ligation reaction performed with benzoic acid (**15**) or cinnamic acid (**18**), and benzyl azide (**16**) or 4-azidoanisole (**20**) and compound **8**-red as catalyst.

The most functionalized material, compound **8**, was finally tested as organocatalyst in a Staudinger ligation of carboxylic acids and azides being inspired by work of Ashfeld and co-workers [24]. In this work the reaction between a carboxylic acid and an organic azide, to afford the corresponding amide is catalyzed by PPh_3_ (10 mol %). The process is general and affords high yields. The catalytic cycle is guaranteed by the presence of PhSiH_3_ that reduces the triphenylphosphine oxide formed to the starting phosphine. In our experiments we substituted triphenylphosphine with the reduced form of compound **8**, **8**-red (see Scheme 5).

As a matter of fact, the reactions reported in Scheme 5 were successful and afforded the expected amides **17** and **19** and **21** in acceptable yields. For a correct comparison with the higher yields reported for the reaction performed in homogeneous phase (94%, 95% and 80%, respectively) it should be stressed that in these experiments the amount of phosphine used is one order of magnitude lower (1% calculated on the basis of the P loading in compound **8**). Further experiments aimed to evaluate the action of the catalyst in new reaction cycles revealed a fast degradation of the efficiency: the yield of amide **17** dropped to 30% and 20% in the second and third cycles while no conversion was observed in the second cycle for amide **19**. The yield of compound **21** was 48% in the second cycle.

## Conclusion

In conclusion, we developed a simple procedure for the covalent decoration of oxidized multi-walled carbon nanotubes and graphene-based materials with three different TPPO derivatives. Materials were completely characterized by FTIR, Raman, XPS spectroscopy and TEM, the loading of phosphorus were quantified by ICP-AES. The higher loading was obtained with the nitrene cycloaddition on CNTs but good results were also obtained with graphene. The reduction of adduct to the correspondent TPP was also investigate, the reduction was confirmed by XPS, whose spectra showed the complete disappear of the phosphine oxide peak and the presence of the intense phosphine peak in 48 h. The possibility to use the TPP group for further modification as binding of Pd nanoparticles, oxidation to phosphine sulfide and selenide are actually under investigation in our laboratory. More significantly, we have explored the ability of one of these materials (the one with the highest loading in phosphine oxide moiety, compound **8**) as heterogeneous catalyst in a Staudinger ligation reaction. Despite the process is still to be optimized, concerning the yield and the recycling of the catalyst, the very low amount of phosphine oxide employed make this approach promising for the development of efficient nanostructured materials useful in organocatalysis.

## Experimental

### Materials

MWCNTs were purchased from Sigma-Aldrich reagent, O.D. × L.= 6–9 nm × 5 µm, carbon >95%, CoMoCat^©^. GPs were supplied from Nanesa in dry powder or water paste, C/O ratio 44:1, carbon >97%, average flake thickness 10 nm (30 layers), average lateral size 10–50 µm. All the other reagents, whose synthesis is not described, were commercially available and have been used without any further purification, if not specified otherwise. Rf values are referred to TLC on silica gel plate (0.25 mm, Merck silica gel 60 F_254_). NMR spectra were recorded on Varian Gemini 200 MHz or Varian Mercury 400 MHz at room temperature. Chemical shifts were reported in parts per million (ppm) relative to the residual solvent peak rounded to the nearest 0.01 for proton and 0.1 for carbon (reference: CHCl_3_ [^1^H:7.26, ^13^C:77.2], DMSO [^1^H:2.5, ^13^C:39.5], MeOH [^1^H:3.35, ^13^C:49.3]. Coupling constants *J* were reported in Hz to the nearest 0.01 Hz. Peak multiplicity was indicated by s (singlet), d (doublet), t (triplet), q (quartet), m (multiplet) and br (broad signal). IR spectra were recorded on a Perkin-Elmer FT-IR 881 or Shimadzu FT-IR 8400s spectrometer. IR data are reported as frequencies in wavenumbers (cm^−1^). UV–vis spectra were recorded on Varian Cary 4000 UV–vis spectrophotometer using 1 cm cell. Fluorescence spectra were registered on a Jasco FP750 spectrofluorimeter using 1 cm cell. Raman spectra were measured by a Renishaw RM2000 instrument with a diode laser emitting at 785 nm. Elemental analyses were performed with a Thermofinnigan CHN-S Flash E1112 analyzer. ICP analysis were made using an Optima 2000 Perkin Elmer Inductively Coupled Plasma (ICP) Dual Vision instrument after acidic mineralization. TEM images were acquired at the electronic microscopic center CNR Florence (CE.M.E.) with a Philips CM12 with CRYO-GATAN UHRST 3500 technology, digital camera and EDAX microanalysis.

#### Synthesis of (4-aminophenyl)diphenylphosphine oxide **1** and (4*-*ethynylphenyl)diphenylphosphine oxide **3**

Compounds **1** [38] and **3** [39] were synthesized by literature procedures in 64% and 72% yield, respectively.

#### Synthesis of (4-azidophenyl)diphenylphosphine oxide **2**

A solution of (4-aminophenyl)diphenylphosphine oxide (**1**, 1.06 g, 3.61 mmol) in acetone (10 mL), H_2_SO_4_ (2.7 mL) and H_2_O (14.4 mL) was added with a solution of NaNO_2_ (0.368 g, 5.33 mmol) in H_2_O (2.2 mL) at 0 °C. After stirring for 1.5 h at 0 °C, a solution of NaN_3_ (0.4 g, 6.13 mmol) in H_2_O (2 mL) was added dropwise at 0 °C. The resulting suspension was stirred for 1.5 h at 0 °C and at room temperature for 15 h. After the completion of reaction, the mixture was extracted with EtOAc (100 mL). The combined organic extracts were washed with brine, dried over anhyd. MgSO_4_, filtered, and evaporated in vacuo to afford azide **2** as an off-white solid (1.09 g, 96%). Mp 119–121 °C; ^1^H NMR (500 MHz, chloroform-*d*) δ 7.76–7.61 (m, 6H), 7.60–7.53 (m, 2H), 7.52–7.44 (m, 4H), 7.17–7.05 (m, 2H); ^13^C NMR (126 MHz, chloroform-*d*) δ 143.98 (d, *J* = 3.0 Hz), 133.85 (d, *J* = 10.8 Hz), 132.29 (d, *J* = 105.0 Hz), 132.08 (d, *J* = 3.2 Hz), 132.01 (d, *J* = 10.1 Hz), 128.80 (d, *J* = 106.9 Hz), 128.58 (d, *J* = 12.1 Hz), 119.10 (d, *J* = 13.0 Hz); ^31^P NMR (202 MHz, chloroform-*d*) δ 28.61. Anal.: calcd for C_18_H_14_N_3_OP: C, 67.71; H, 4.42; N, 13.16%; found: C, 67.66; H, 4.48; N, 13.22%.

#### Synthesis of *p*-azidoaniline **10**

Compound **10** was synthesized from 4-bromoaniline in quantitative yield following a procedure reported in literature [40].

#### Oxidation of CNTs

To a 100 mL flask were added CNTs 500 mg and 40 mL of a 3:1 solution of 96% sulfuric acid/65% nitric acid. The mixture was stirred at reflux for 30 min, diluted with fresh water in ice bath and the acidic solution removed by centrifugation (10 min at 1400 rcf). The solid was dispersed with water and centrifuged 30 min at 1400 rcf. The solid was again dispersed with water, filtered over a 0.2 µM PC membrane and washed until the filtered solution exhibited a neutral pH. Purified ox-MWCNTs were collected. Yield 39% (194.6 mg); FTIR: 3431, 1704, 1224 cm^−1^; Elemental analysis C, 77.72; H, 0.84; N, 0.19%.

#### Synthesis of CNTs-Tour-PO (**6**)

To a 5 mL flask were added CNTs (10 mg, 0.83 mmol) and (4-aminophenyl)diphenylphosphine oxide (**1**, 54.3 mg, 0.185 mmol and 2.5 mL of anhydrous DMF. The mixture was kept in an ultrasound bath for 10 min, under inert atmosphere, and 19.3 mg (0.165 mmol) of isopentyl nitrite were added. The mixture was stirred at 80 °C under nitrogen for 16 h. The dispersion was diluted a 1:1 solution of isopropanol/diisopropyl ether and centrifuged for 15 min at 1400 rcf. The supernatant solution was removed and the solid was washed by 5 cycles of dispersion in fresh solvent and centrifugation (4 times with a 2:1 isopropanol/diisopropyl ether solution and once with a 1:1 solution of the same solvents. The product was finally recovered and dried to afford 15 mg of a black powder. ICP-AES analysis: phosphorus 1.255%, 0.41 mmol/g. FTIR (KBr): 3328, 1724, 1579, 1384, 1154 and 1116 cm^−1^.

#### Synthesis of GPs-Tour-PO (**7**)

To a 5 mL flask were added GPs 11.6 mg (0.95 mmol), (4-aminophenyl)diphenylphosphine oxide (**1**, 54.3 mg, 0.185 mmol) and 2.5 mL of anhydrous DMF. The mixture was kept in an ultrasound bath for 30 min, under inert atmosphere, then 19.3 mg (0.165 mmol) of isopentyl nitrite were added. The mixture was stirred at 80 °C under nitrogen for 16 h. The dispersion was diluted with isopropanol and centrifuged for 15 min at 1400 rcf. The supernatant was removed. The solid was washed by cycle of dispersion in fresh methanol and centrifugation (six times). The product was recovered and dried (12.5 mg). ICP-AES analysis: phosphorus 0.275%, 0.087 mmol/g.

#### Synthesis of ox-MWCNTs-nitrene-PO (**8**)

To a 50 mL flask were added CNTs (10 mg) and 20 mL of 1,2-dichlorobenzene. The mixture was kept in an ultrasound bath for 30 min. (4-Azidophenyl)diphenylphosphine oxide **2** (39 mg 0.12 mmol) was added and the dispersion sonicated again for 10 min. The resulting dispersion was kept at 165 °C under vigorous stirring for 4 days. The mixture was filtered through a 0.2 µm pore PTFE membrane and thoroughly washed with a solution of diisopropyl ether and isopropanol 1:1. The product was recovered and dried to afford 13.3 mg of a black powder. Elemental analysis C, 76.07; H, 1.12; N, 0.82%. ICP-AES phosphorus 1.807%, 0.583 mmol/g. FTIR (KBr): 3312, 1718, 1559, 1164 and 1114 cm^−1^ P=O stretching.

#### Synthesis of GPs-nitrene-PO (**9**)

To a 50 mL flask were added GPs (11.5 mg) and 20 mL of 1,2-dichlorobenzene. The mixture was kept in an ultrasound bath for 30 min. (4-Azidophenyl)diphenylphosphine oxide **2** (39 mg, 0.12 mmol) was added and the mixture sonicated again for 10 min. The dispersion was kept at 165 °C under vigorous stirring for 4 days. The mixture was then diluted with isopropanol and centrifuged 15 min at 1400 rcf. The supernatant was removed and the precipitate was washed by 5 cycles of dispersion and centrifugation (5 min in ultrasound bath and centrifugation for 15 min at 1400 rcf), with a solution of diisopropyl ether and isopropanol 1:1. The product was recovered and dried to afford 13.1 mg. Elemental analysis C, 90.36%; H, 0.58; N, 0.42%. ICP-AES 0.61% of phosphorous. FTIR (KBr): 1195 and 1181 cm^−1^ P=O stretching.

#### Synthesis ox-MWCNTs-N_3_ (**11**)

To a 10 mL flask were added CNTs (50 mg), **10** (122.96 mg, 0.91 mmol), dry DMF (5.2 mL) and the mixture was sonicated in an ultrasound bath (10 min). To the dispersion was added isopentyl nitrite 97.62 mg (0.83 mmol) was added and the mixture was stirred at 60 °C for 24 h. The suspension was filtered over a 0.2 μm PTFE membrane, and the solid was washed with DMF and acetone until colorless solution obtained. CNTs-Azide were recovered with acetone and dried to afford 50.5 mg of a black powder. FTIR (KBr): 2118 nm N_3_ stretching. Elemental analysis C, 66.94%; H, 3.83%; N, 7.00%. Azide loading based on elemental analysis 1.63 mmol/g.

#### Synthesis of GPs-N_3_ (**12**)

To a 25 mL flask were added GPs (33 mg), **10** (82 mg 0.61 mmol), 1,2-dichlorobenzene (2.64 mL) and dry DMF (5.2 mL). The mixture was dispersed in an ultrasound bath. Then, isopentyl nitrite (63.9 mg, 0.54 mmol) was added and the mixture was stirred at 60 °C for 24 h. The suspension was filtered over a 0.2 μm ptfe membrane. Thereafter, the solid was thoroughly washed with DMF and acetone until a colorless solution was obtained. The material was recovered with acetone and dried to obtain 32.5 mg of a black powder. FTIR (KBr) 2119 nm N_3_ stretching. Elemental analysis C, 97.7%; H, 0.15%; N, 0.31%. Azide loading based on elemental analysis 0.075 mmol/g.

#### Synthesis of CNTs-Click-PO (**13**)

To a 25 mL flask were added **11** (30 mg, 0.049 mmol of azide), phosphine oxide **3** (17.75 mg, 0.059 mmol, 1.2 equiv) of copper iodide triethyl phosphite (3.49 mg, 0.0098 mmol, 0.2 equiv) and 8.5 mL of degassed dry DMF. The mixture dispersed in an ultrasound bath under inert atmosphere. After dispersion, DIPEA 18.35 mg (0.142 mmol, 2.9 equiv) was added and the mixture was stirred at 60 °C for 48 h. The suspension was filtered over a 0.2 μm PTFE membrane and the solid was thoroughly washed with DMF and acetone until a colorless solution was obtained. The material was recovered and dried to afford 28.2 mg of a black powder. FTIR (KBr) 3105, 2103, 1722, 1658, 1579, 1386, 1170 and 1118 cm^−1^. ICP AES analysis: phosphorus 0.847%.

#### Synthesis of GPs-Click-PO (**14**)

To a 25 mL flask were added with **12** (28.5 mg, 0.0023 mmol of azide), phosphine oxide **3** (1 mg, 0.0027 mmol, 1.2 equiv) copper iodide triethyl phosphite (0.162 mg, 0.00045 mmol, 0.2 equiv) and degassed 1,2-dichlorobenzene (8.5 mL). The mixture was dispersed in an ultrasound bath. DIPEA (1 mg, 0.0078 mmol, 2.9 equiv) was added and the reaction mixture was stirred at 60 °C for 48 h. The suspension was diluted with acetone and centrifuged (10 min at 1500 rcf) to remove 1,2-dichlorobenzene. Then the solid was dispersed in DMF and filtered over a 0.2 μm PTFE membrane. The solid was washed with DMF and acetone until colorless solution obtained. The material was recovered and characterized to afford 28.7 mg of a black powder. ICP AES analysis: phosphorus 0.057%.

#### General procedure for the reduction of PO

To a Pyrex tube was added to carbonaceous substrate (5 mg), degassed 1,2-dichlorobenzene (1 mL) and the mixture was dispersed in an ultrasound bath. The mixture was then added with triethyl phosphite (387.6 mg, 2.33 mmol) and trichlorosilane (134.2 mg, 0.99 mmol) and the reaction left 48 h at 100 °C under vigorous stirring. The material was washed by repeated dispersion and centrifugation cycles (10 min at 1400 rcf) one cycle with isopropanol to remove the reaction solvent, three cycles with 1 M aqueous sodium hydroxide, three cycles with aqueous hydrogen chloride 0.1 M, three cycles with methanol and three cycles with isopropyl ether. The reduction was checked by XPS spectroscopy.

#### General procedure for the Staudinger ligation

To a pirex tube were added with phosphine decorated CNTs (**8**-red, 5 mg) degassed dry toluene (1 mL), carboxylic acid (0.19 mmol, 1 equiv), a solution of benzyl azide (0.19 mmol) in 0.5 mL of degassed toluene and phenylsilane (21 mg, 0.19 mmol, 1 equiv) and the reaction was stirred at 110 °C under nitrogen atmosphere for 22 h. The catalyst was recovered by centrifugation (2 × 15 min at 1500 rcf) and dispersion with toluene 20 mL. The solution was evaporated under vacuum giving the crude product, the amide was recovered after flash chromatography (silica) using a mixture of hexane/ethyl acetate 1:1.

*N*-Benzylbenzamide (**17**): *R*_f_ = 0.48 (hexane/ethyl acetate 1:1), yield 75%. Spectral data already reported in literature [24].

*N*-Phenylcinnamamide (**19**): *R*_f_ =0.77 (hexane/ethyl acetate 1:1), yield 55%. Spectral data already reported in literature [24].

*N*-Phenylcinnamamide (**21**): *R*_f_ =0.64 (hexane/ethyl acetate 1:1), yield 58%. Spectral data already reported in literature [24].

## Supporting Information

File 1 IR, Raman and XPS spectra, NMR spectra of amides **17**, **19** and **21**, GC analysis of **17** and **21**.
