# Supplementary material for: Nanostructured carbon materials decorated with organophosphorus moieties: synthesis and application
Source: Beilstein J Nanotechnol. 2017 Feb 22;8:485–93. doi: 10.3762/bjnano.8.52 (PMC5331327; doi:10.3762/bjnano.8.52)
Supplement: File 1 — IR, Raman and XPS spectra, NMR spectra of amides 17, 19 and 21, GC analysis of 17 and 21. [file Beilstein_J_Nanotechnol-08-485-s001.pdf]

Supporting information

for

Nanostructured carbon materials decorated with organophosphorus moieties: synthesis and application

Giacomo Biagiotti[1], Vittoria Langè[1], Cristina Ligi[1], Stefano Caporali[2,3], Maurizio Muniz-Miranda[1], Anna Flis[4], K. Michał Pietrusiewicz[4], Giacomo Ghini[5], Alberto Brandi[1] and Stefano Cicchi[*1]

Address: [1]Dipartimento di Chimica Ugo Schiff Università di Firenze, Via della Lastruccia 3–13, 50019 Sesto Fiorentino, Italy, [2]Consorzio Interuniversitario Nazionale per la Scienza e Tecnologia di Materiali INSTM Firenze 50123, Italy, [3]Istituto dei Sistemi Complessi Consiglio Nazionale delle Ricerche Sesto Fiorentino (FI) 50019, Italy, [4]Department of Organic Chemistry Maria Curie-Skłodowska University ul. Gliniana 33, 20-614 Lublin, Poland and [5]Nanesa S.r.l. Via Setteponti 143 – 1, 52100 Arezzo, Italy

Email: Stefano Cicchi - stefano.cicchi@unifi.it

* Corresponding author

IR, Raman and XPS spectra, NMR spectra of amides 17, 19 and 21, GC analysis of 17 and 21

XPS Measurements

The chemical composition of the near surface sample portion was obtained by means of X-ray Photoelectron Spectroscopy (XPS). The experiments were carried out in an ultrahigh vacuum (UHV, 10-9 mbar) system equipped with a VSW HAC 500 hemispherical electron-

energy analyzer using a non-monochromatic Mg Kα X-ray source operating at 120 W power (10 kV × 10 mA). The samples were introduced in the UHV system via a loadlock under an inert gas (N2) flux. Survey and high-resolution spectra (C1s, O1s, P2p) were acquired in the constant analyzer energy mode (CAE) at pass energy Epas = 22 eV with a step size of 1.0 and 0.1 eV, respectively. The peaks were fitted using CasaXPS software employing Gauss-Lorentz curves after subtraction of a Shirley-type background. Binding Energy (BE) scale was calibrated taking as reference the position of aliphatic C1s component (adventitious carbon) at 284.8 ± 0.1 eV.

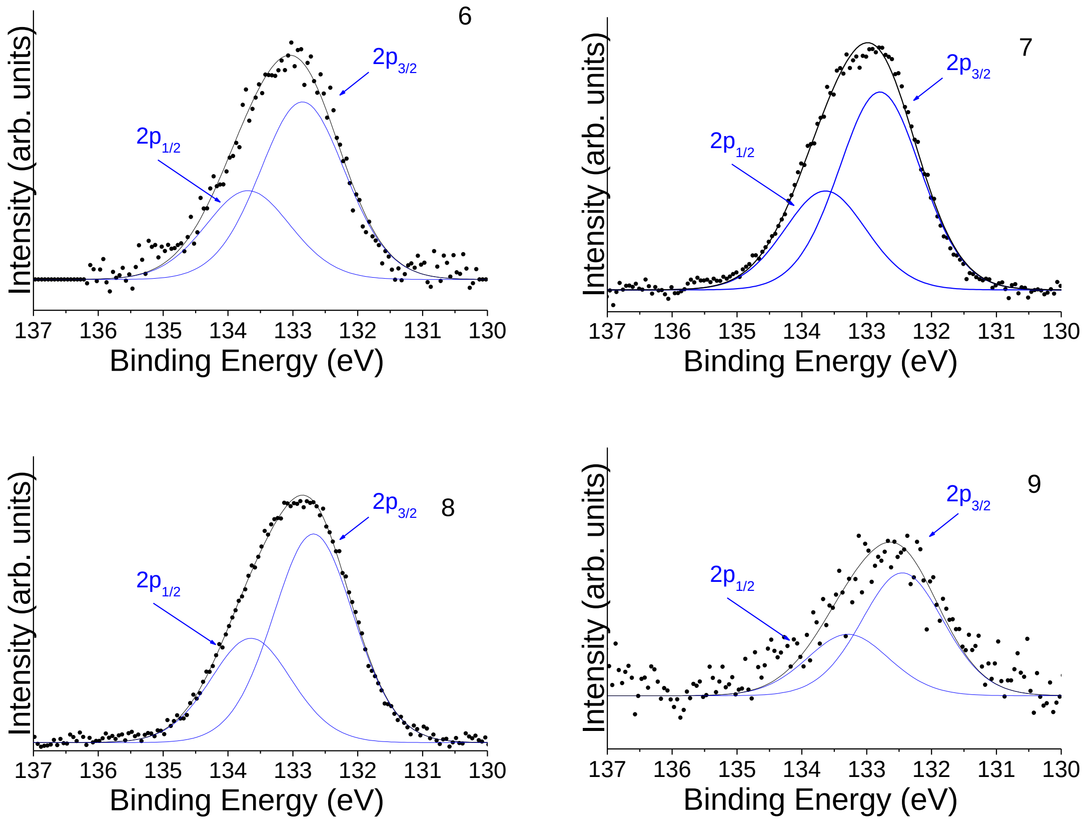

Figure S1: XPS spectra of compounds **6**, **7**, **8**¸ and **9**, all signals are at 132.8 eV.

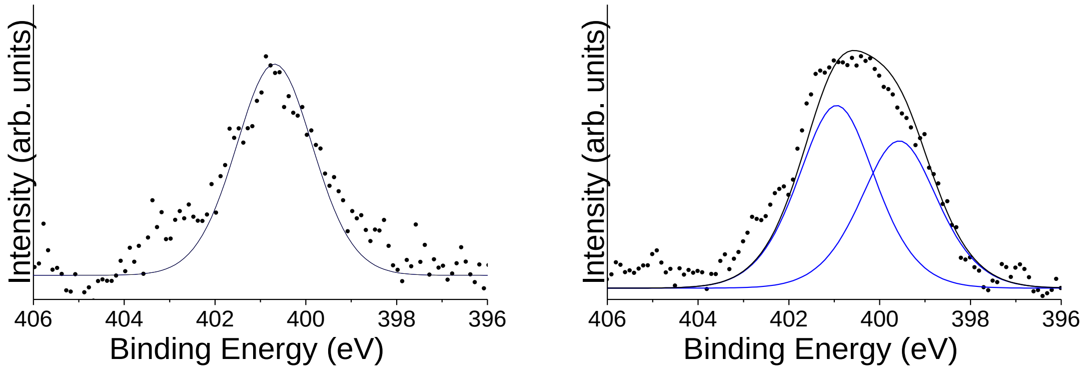

Figure S2: XPS spectra of compounds 6 on the left and 7 on the right, the binding energy of 400.7 eV, which evidence the presence of the nitrogen in both samples.

FT-IR SPECTRA

IR spectra were recorded on a Perkin-Elmer FT-IR 881 or Shimadzu FT-IR 8400s spectrometer All the spectra were recorded in KBr pellets with a concentration of substances of 1mg in 100 mg and under nitrogen atmosphere to remove the noise.

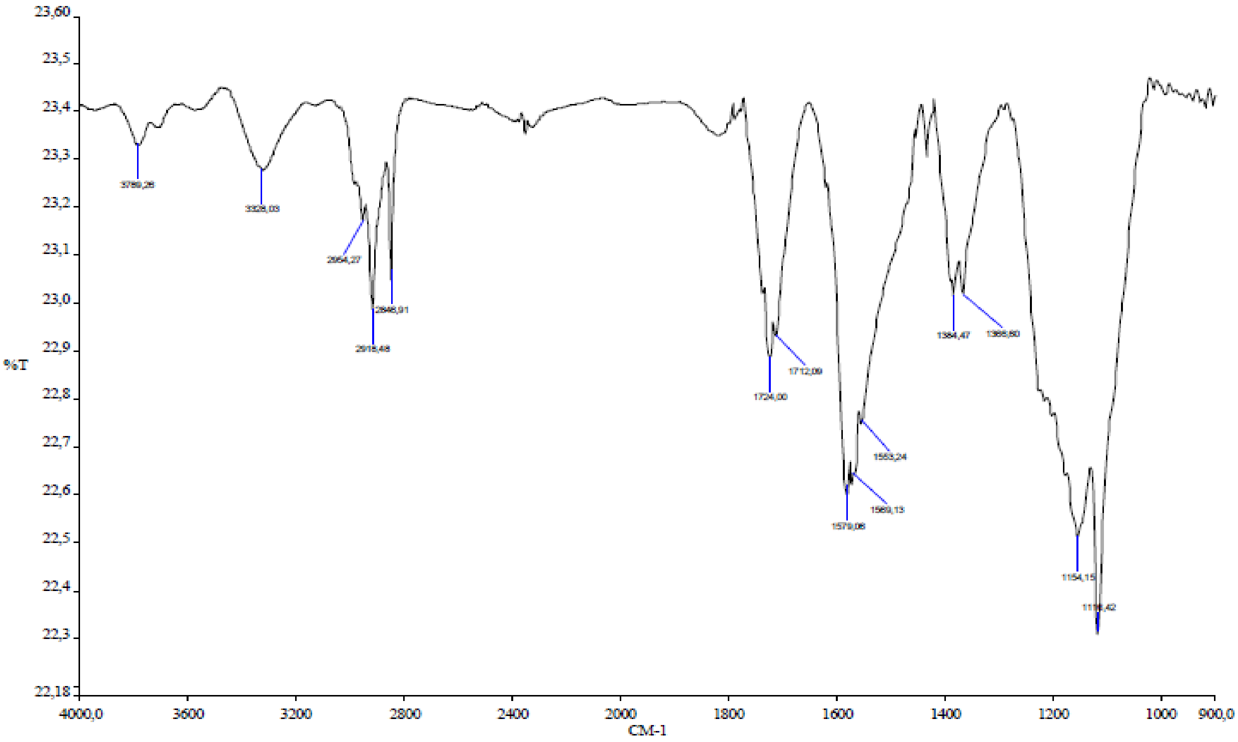

Figure S3: FT-IR Spectrum of Ox-MWCNTs-Tour-PO (6).

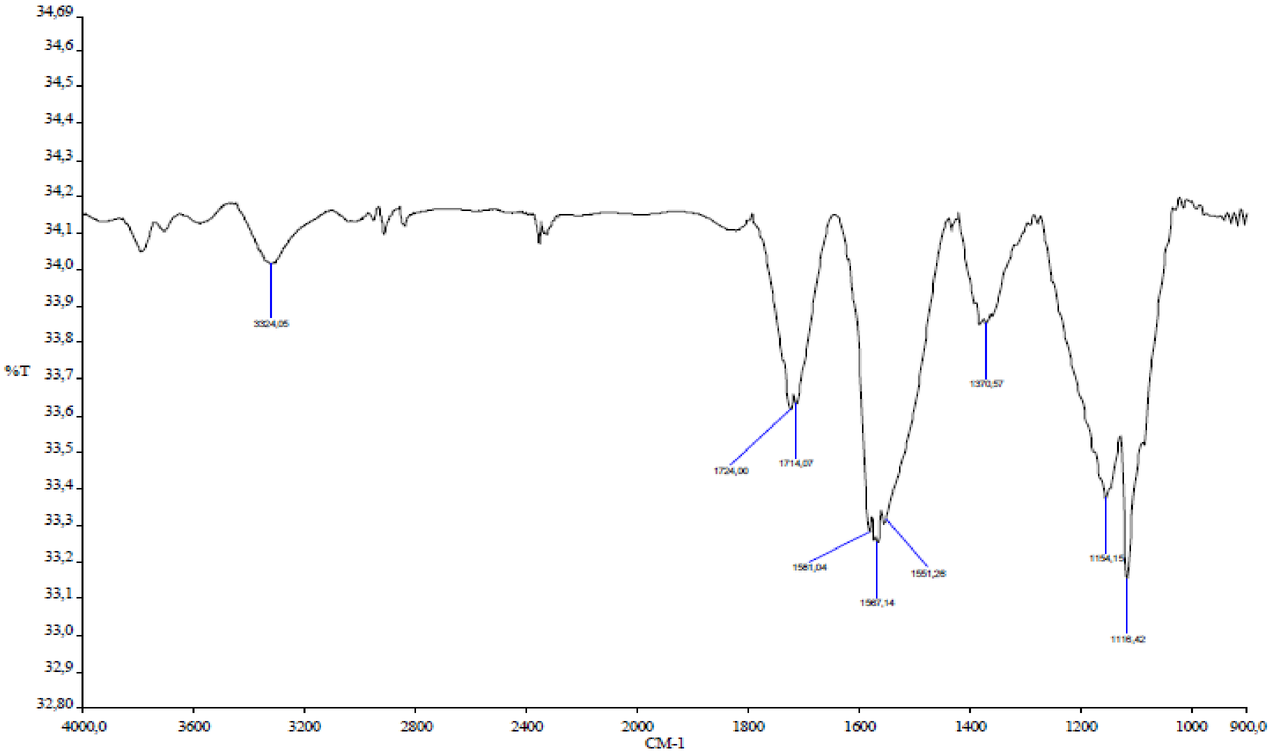

Figure S4: FT-IR Spectrum of CNT-Nit-PO (8).

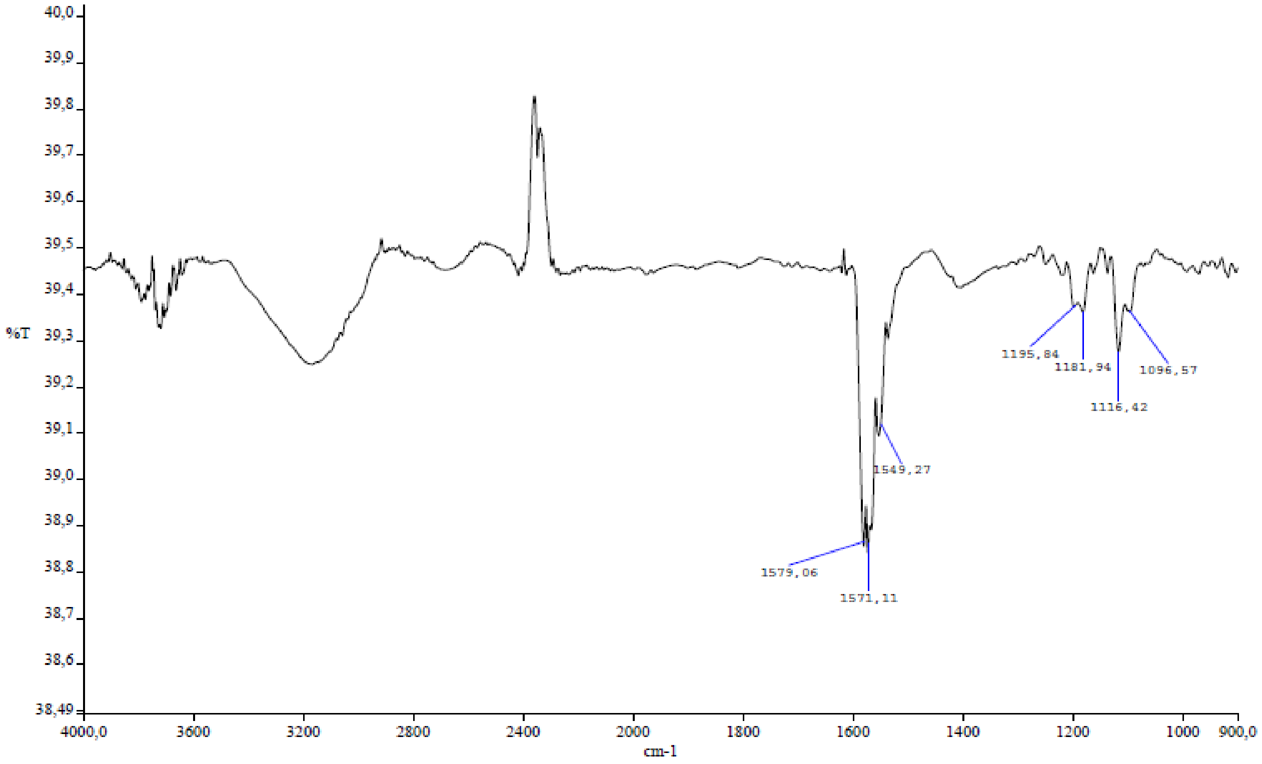

Figure S5: FT-IR spectrum GNP-Nit-PO (9).

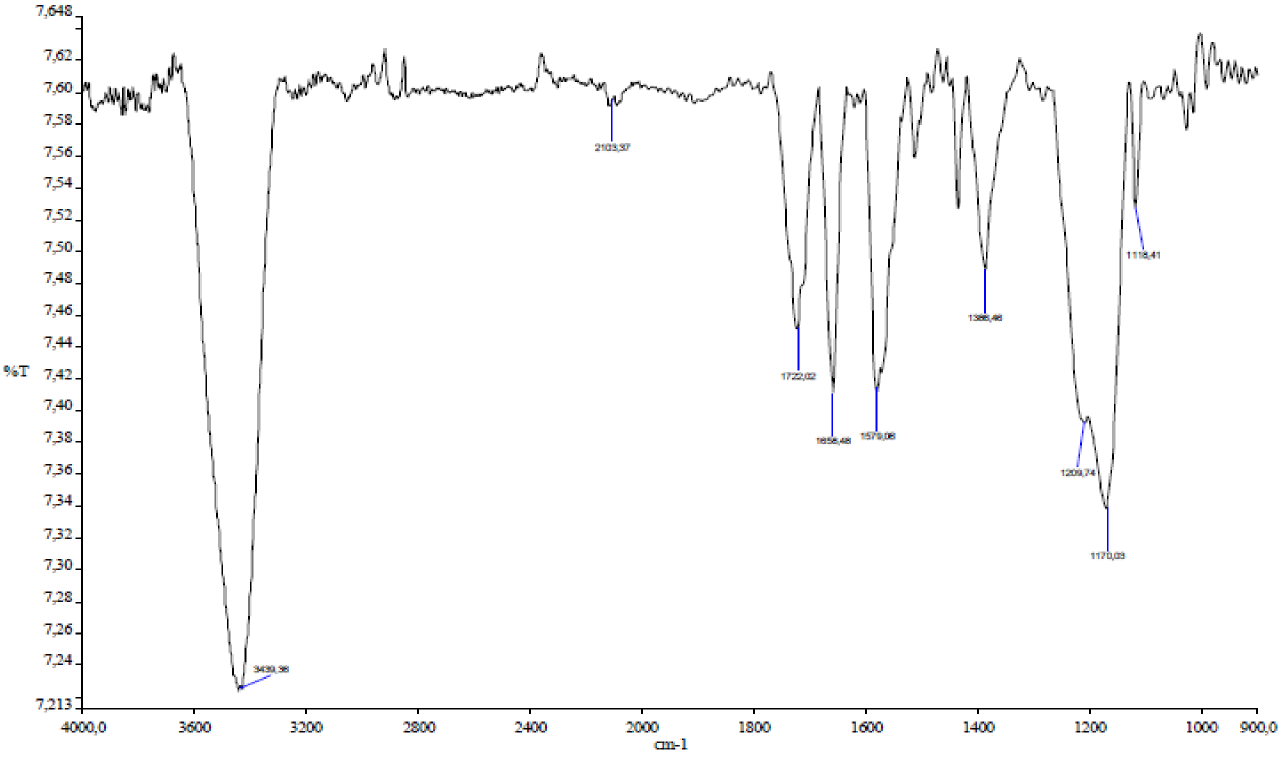

Figure S6: FT-IR spectrum OxMWCNTs-Click-PO (13).

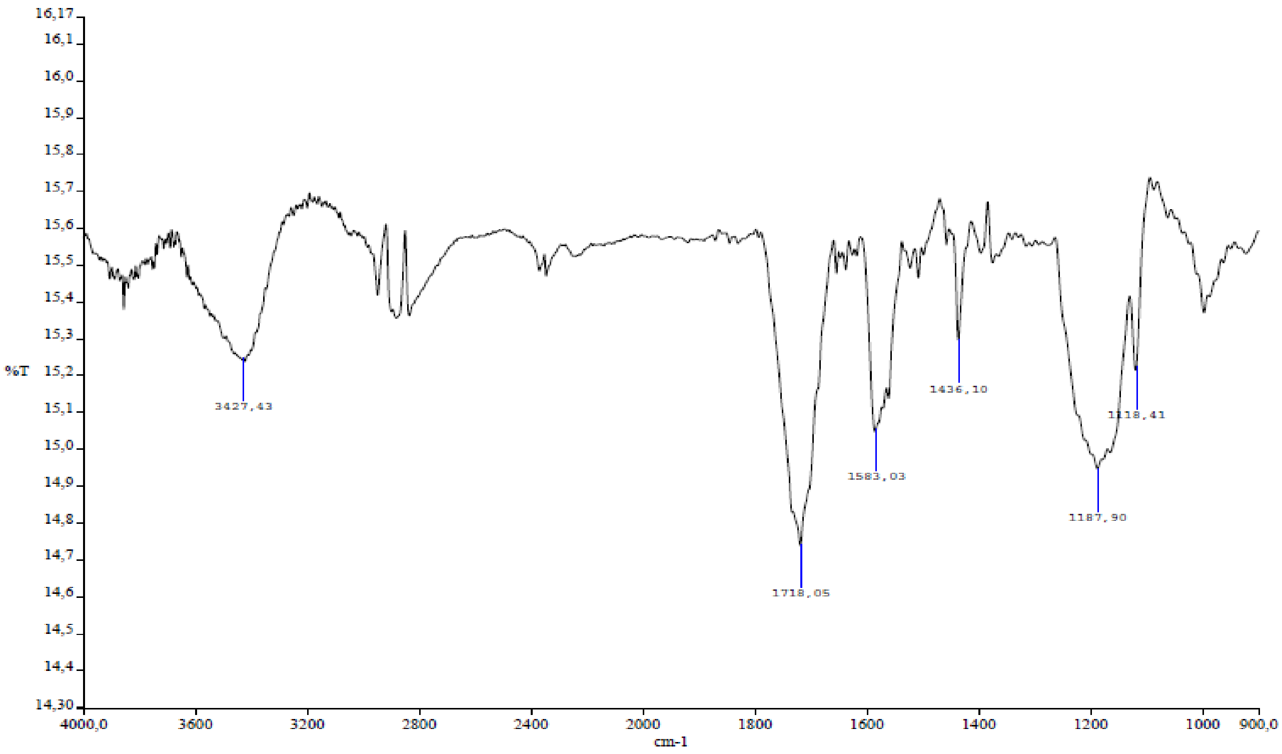

Figure S7: FT-IR spectrum OxMWCNTs-Tour-P (6-red).

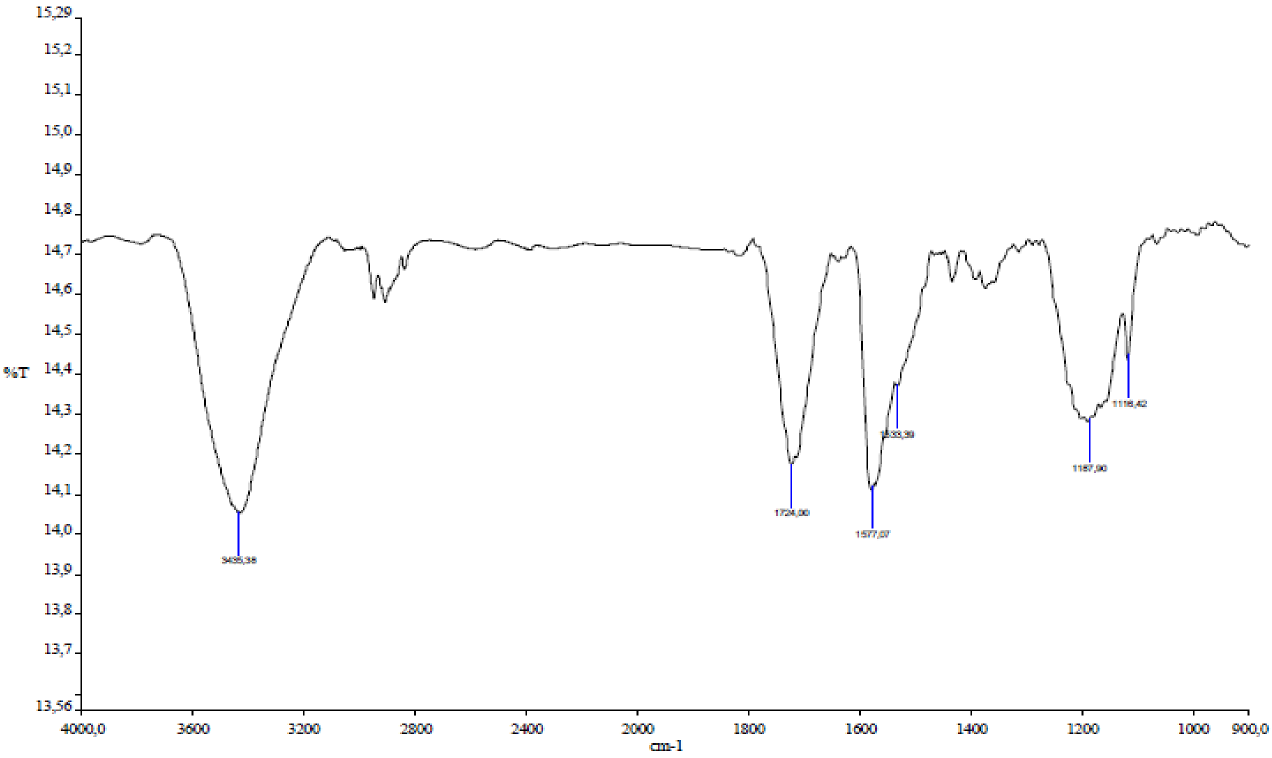

Figure S8: FT-IR spectrum OxMWCNTs-Nit-P (8-red).

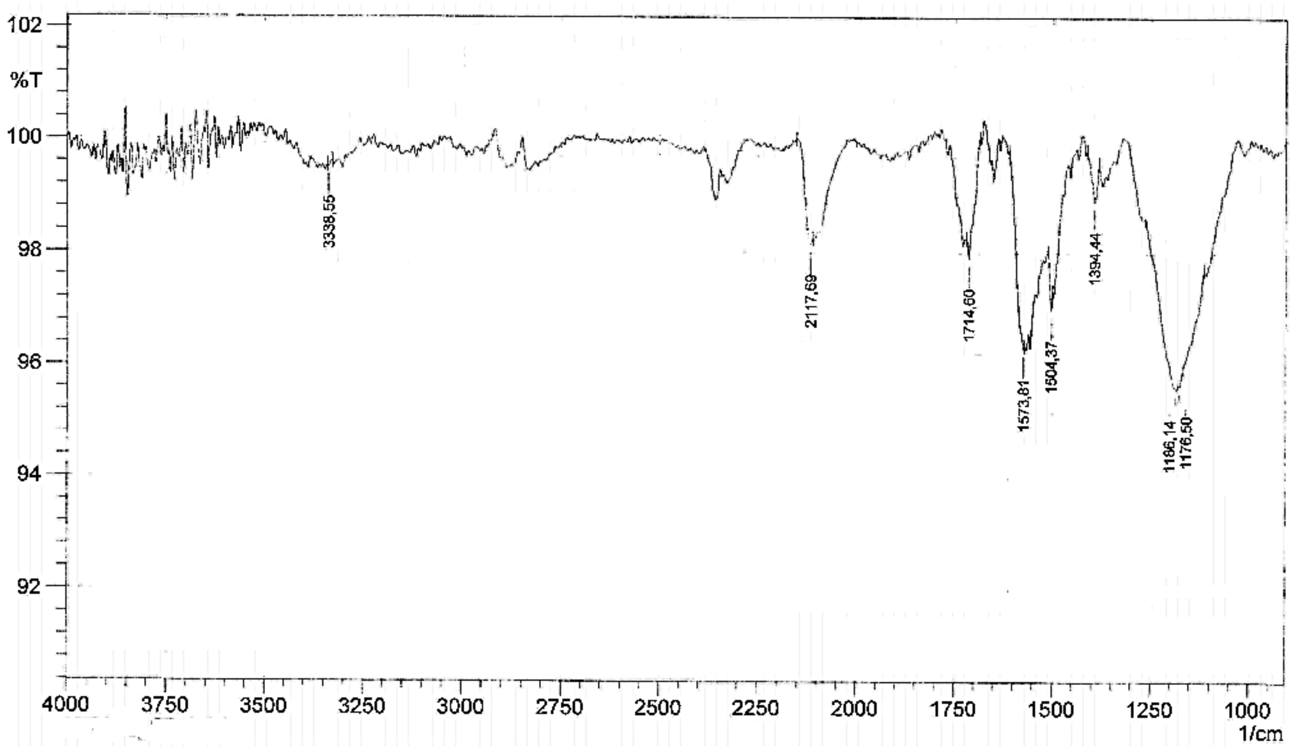

Figure S9: FT-IR spectrum OxMWCNTs-N_3 (11).

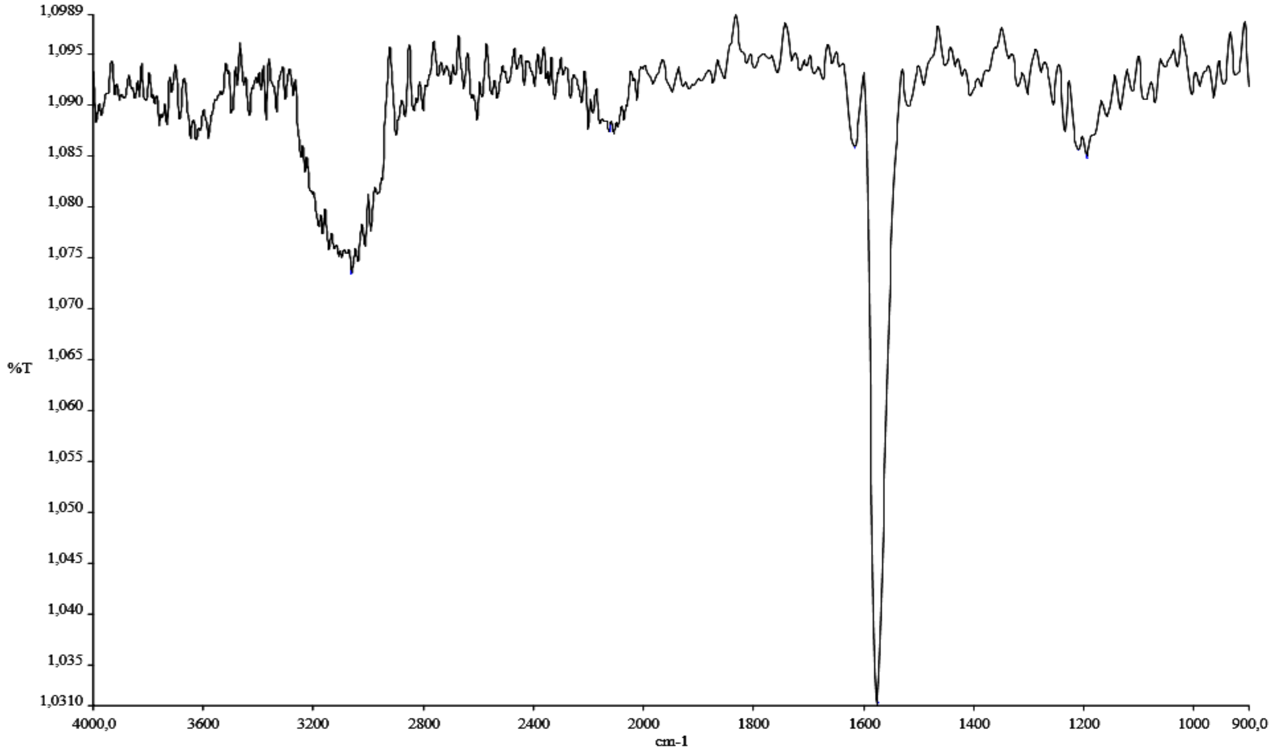

Figure S10: FT-IR spectrum GNPs-N_3 (12).

Raman Measurements

Raman spectra were measured by a Renishaw RM2000 instrument, equipped with a diode laser emitting at 785 nm. Sample irradiation was accomplished by using the 50x microscope objective of a Leica Microscope DMLM. The beam power was ~3mW, and the laser spot size was adjusted between 1 and 3 micrometers. The backscattered Raman signal was fed into the monochromator through 40 micrometer slits and detected by an air-cooled CCD (2.5 cm-1 per pixel) filtered by a double holographic Notch filters system. Spectra were calibrated with respect to a silicon wafer at 520 cm-1.

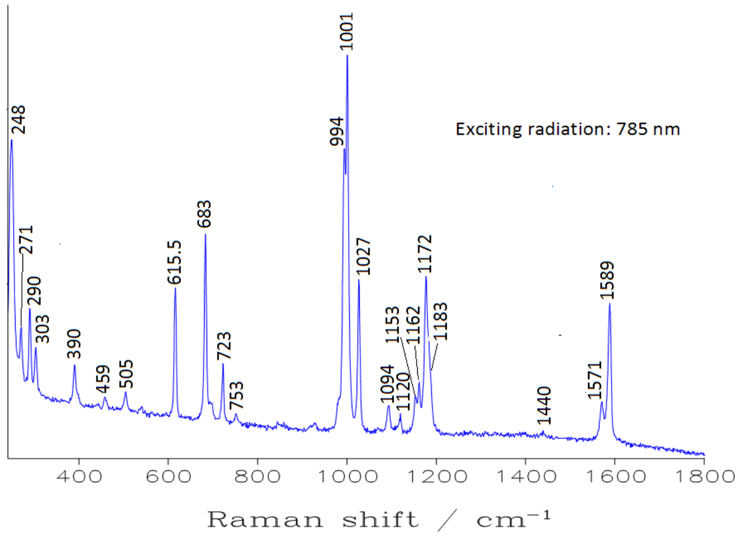

Raman Frequences	Assignment
615.5	ring bending in plane
683	ring bending in plane
994	Ring breathing mode
1001	Ring breathing mode
1027	Ring trigonal mode
1094	P-Ph stretching
1120	P-Ph stretching
1153	H ring bending in plane
1162	H ring bending in plane
1172	P=O stretching
1571	C=C ring stretching
1589	C=C ring stretching

Figure S11: On the left the raman spectrum of phosphine oxide 2, on the right the signal assignment.

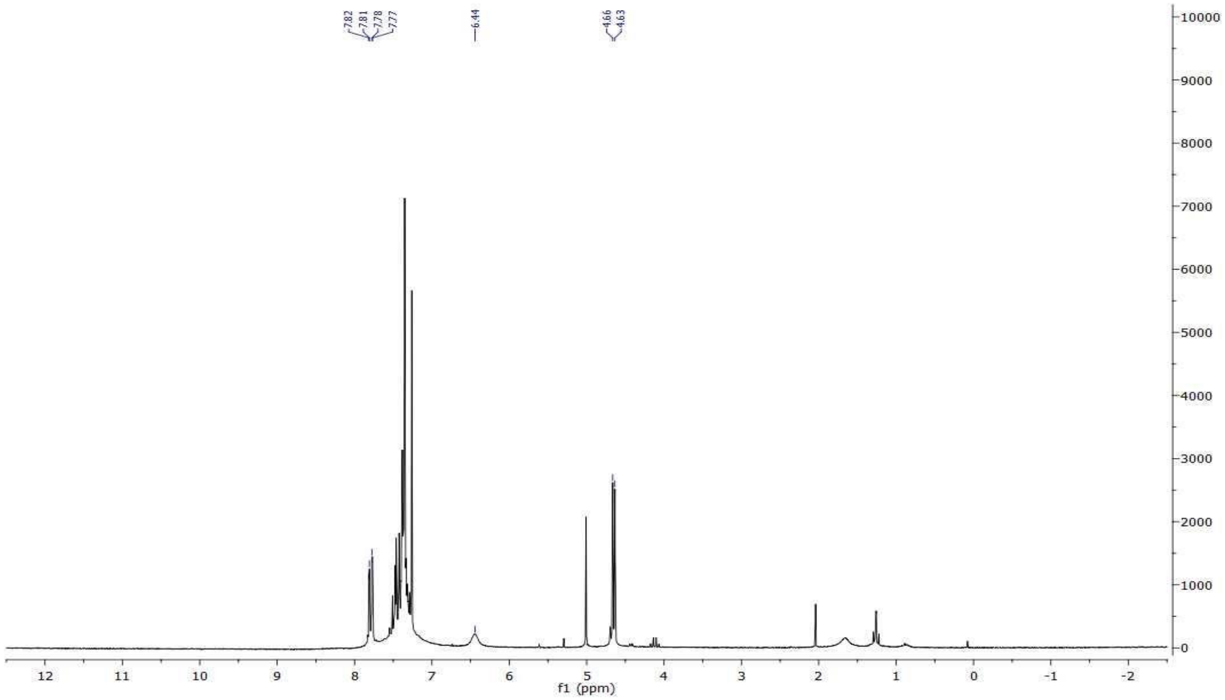

Figure S12: ^{1}H NMR (200 MHz $CDCl_3$) of product **17**.

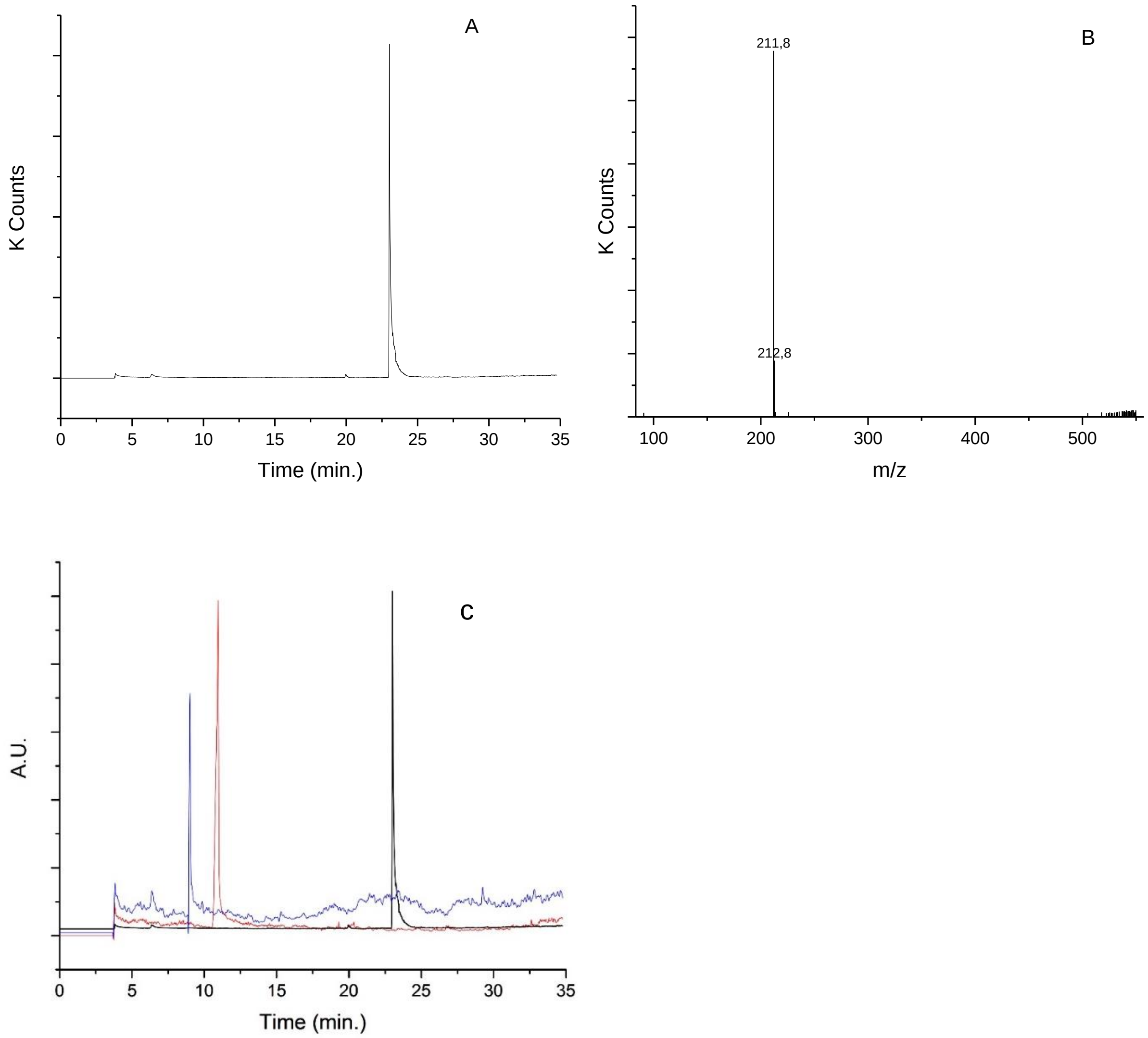

Figure S13: GC-MS Spectra of compound **17**: A) Chromatogram of the purified product; B) Mass spectrum with chemical ionization; C) Comparison three different chromatograms: black product 17, blue benzyl azide and benzoic acid red.

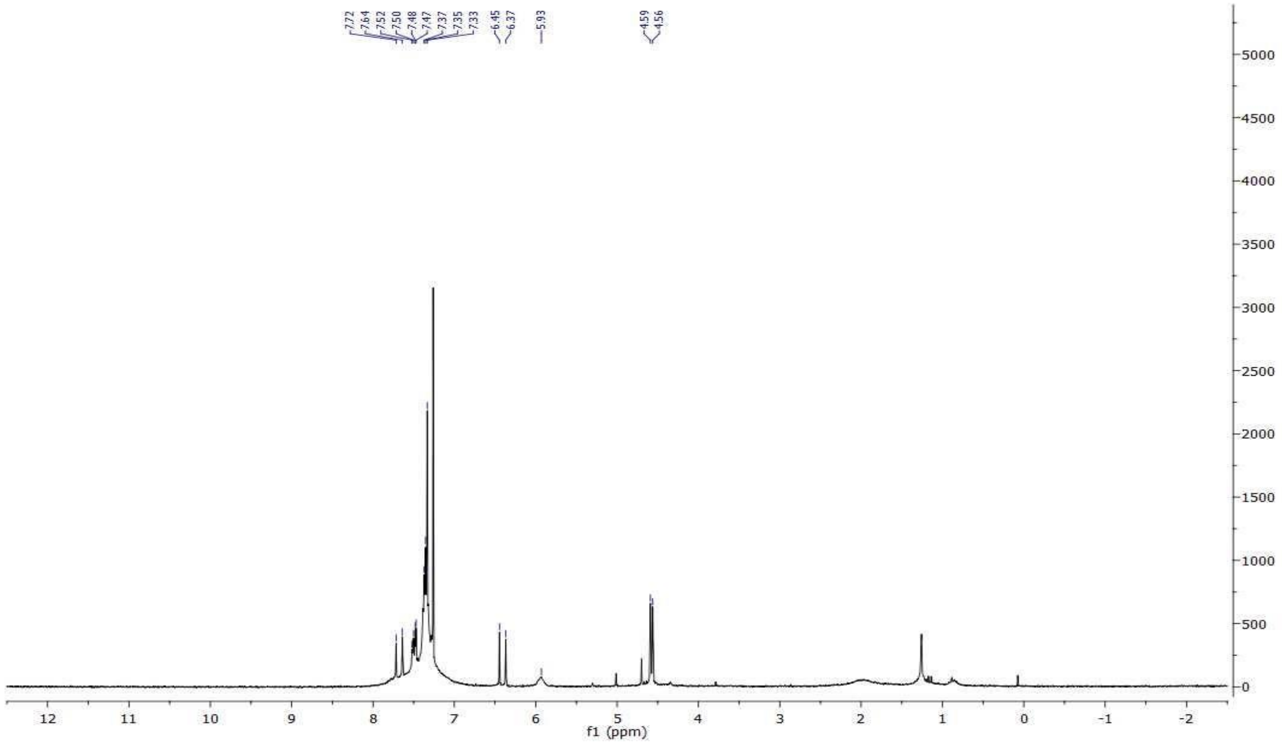

Figure S14: ^{1}H NMR (200 MHz $CDCl_3$) of Product **19**.

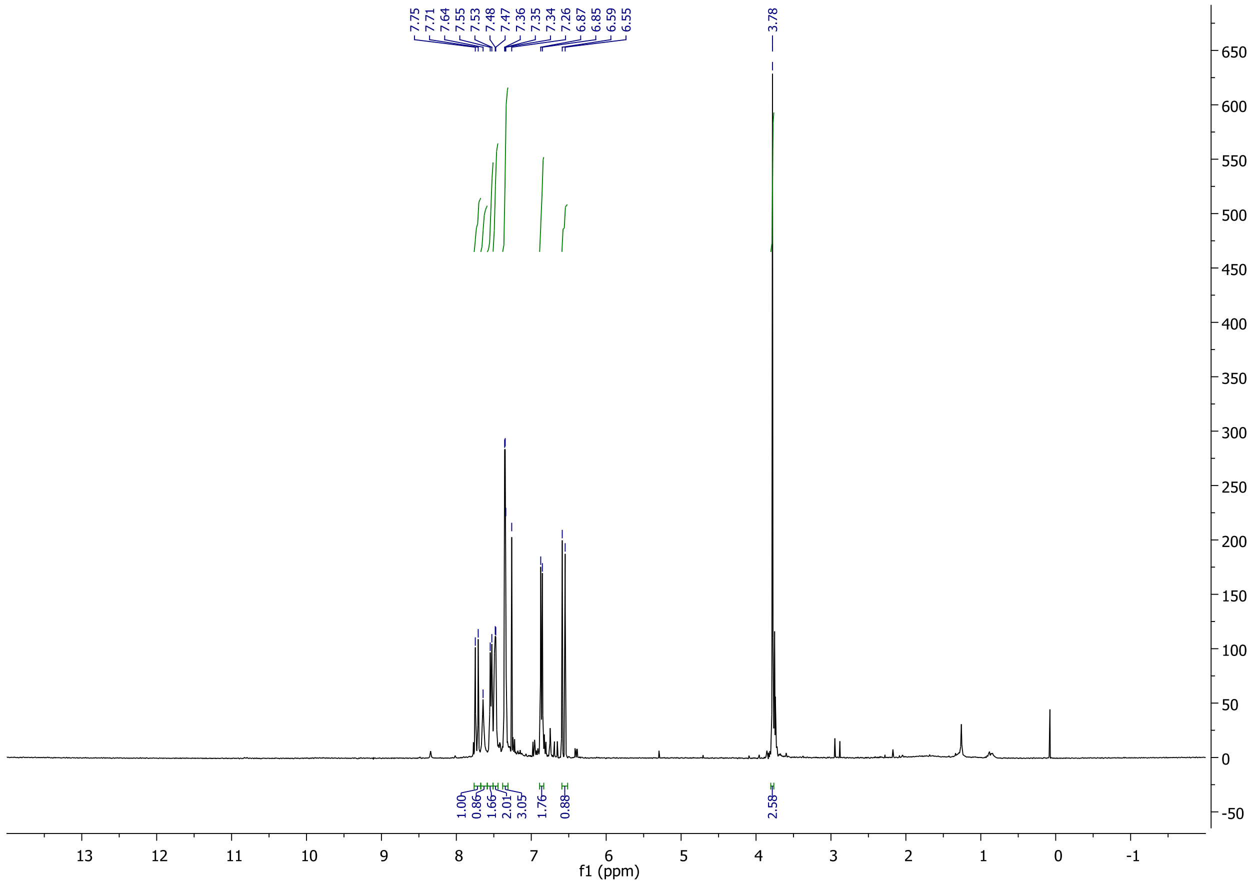

Figure S15: ^{1}H NMR (400 MHz $CDCl_3$) of Product **21**.

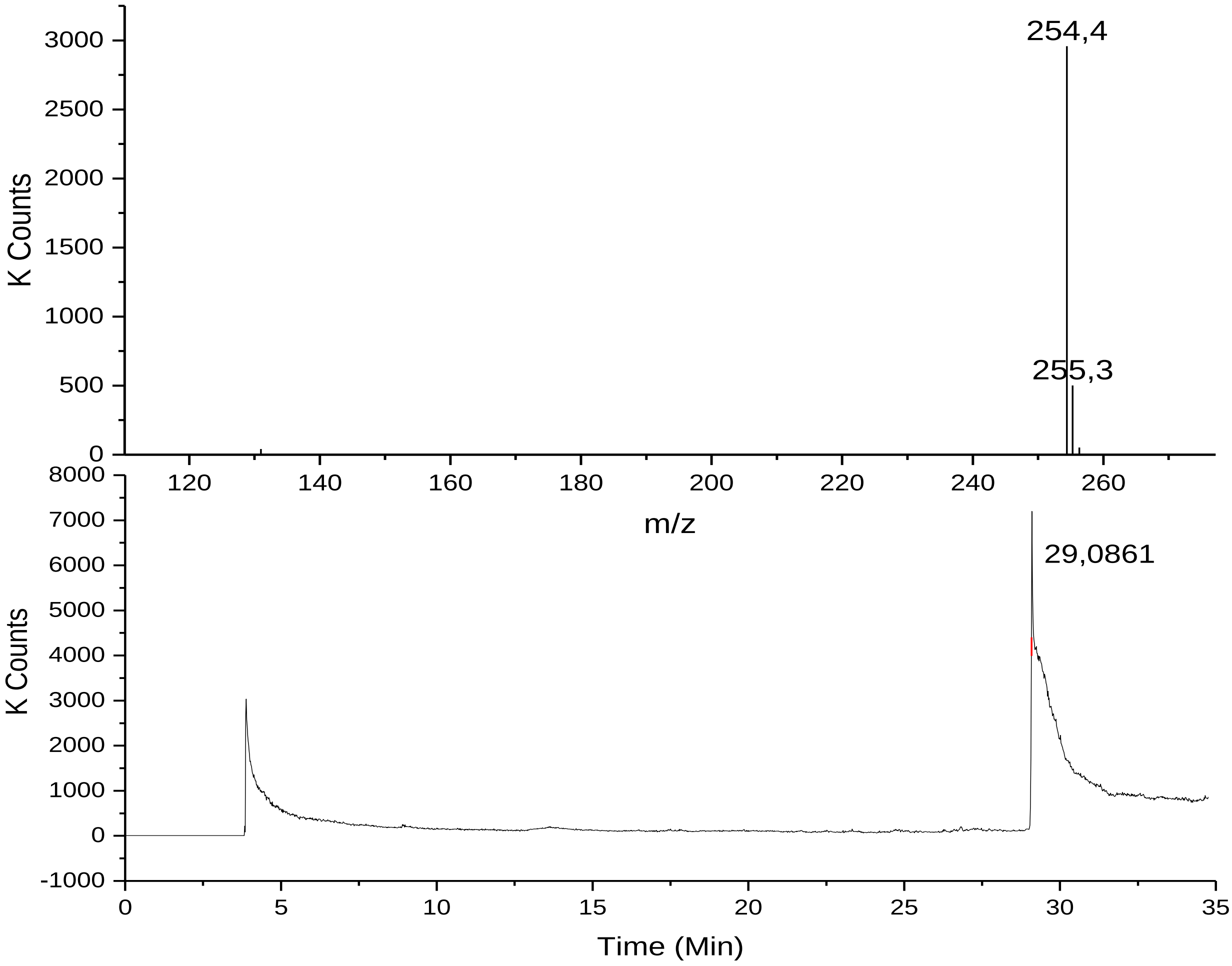

Figure S16: GC-MS Spectra of compound **21**: top Mass spectrum with chemical ionization; bottom chromatogram of the purified product.
